# Sister Bradford's Death

**Published:** 1920-04-03

**Authors:** 


### Sister Bradford's Death.

Miss M. Campbell writes: "On February 5, at the home of her sister, 'Avondale,' Havant, there passed away a very sweet and brave soul, Miss Alice Bradford. Weeks of patient suffering closed a life devoted to the needs of the sick and dying. Many will mourn her who have reason for deep gratitude and love.

"Trained at Winchester Hospital and St. Marylebone Infirmary, she was early imbued with the 'vocation' ideal of a nurse's life—to that high standard she dedicated the future. Deeply religious, her work was her religion, and those who were privileged to work with her could not but be influenced by this.

"Almost the last years of her life were spent in hospital work in Shanghai, and there she contracted the illness which ended fatally. I have known Sister Bradford for many years, first as her probationer, later as fellow-sisters in hospital, and finally, by a strange turn of the wheel of Fate, as her matron. I have had opportunities of knowing her from all points, and it is with much affection and deep gratitude that I can record the beauty of her character, her steadfastness, cheerful optimism—her courage, tact, and loyalty have been indeed an encouragement, example, and often an inspiration. She was in all respects a great nurse. A true friend, as such to be deeply mourned."

